# Reference-free detection of isolated SNPs

**DOI:** 10.1093/nar/gku1187

**Published:** 2014-11-17

**Authors:** Raluca Uricaru, Guillaume Rizk, Vincent Lacroix, Elsa Quillery, Olivier Plantard, Rayan Chikhi, Claire Lemaitre, Pierre Peterlongo

**Affiliations:** 1 University of Bordeaux, CNRS/LaBRI, F-33405 Talence, France; 2 University of Bordeaux, CBiB, F-33000 Bordeaux, France; 3 INRA, UMR1349 IGEPP, Le Rheu, France; 4 GenScale, INRIA Rennes Bretagne-Atlantique, IRISA, Rennes, France; 5 BAMBOO, INRIA Grenoble Rhone-Alpes, Lyon, France; 6 Laboratoire de Biométrie et Biologie Évolutive, Université Lyon 1 UMR CNRS 5558, Lyon, France; 7 INRA, UMR1300 Biology, Epidemiology and Risk Analysis in Animal Health, Nantes, France; 8 LUNAM University, Oniris, Nantes Atlantic College of Veterinary Medicine and Food Sciences and Engineering, UMR BioEpAR, Nantes, France; 9 Department of Computer Science and Engineering, The Pennsylvania State University, University Park, PA, 16802, USA

## Abstract

Detecting single nucleotide polymorphisms (SNPs) between genomes is becoming a routine task with next-generation sequencing. Generally, SNP detection methods use a reference genome. As non-model organisms are increasingly investigated, the need for reference-free methods has been amplified. Most of the existing reference-free methods have fundamental limitations: they can only call SNPs between exactly two datasets, and/or they require a prohibitive amount of computational resources. The method we propose, discoSnp, detects both heterozygous and homozygous *isolated* SNPs from any number of read datasets, without a reference genome, and with very low memory and time footprints (billions of reads can be analyzed with a standard desktop computer). To facilitate downstream genotyping analyses, discoSnp ranks predictions and outputs quality and coverage per allele. Compared to finding isolated SNPs using a state-of-the-art assembly and mapping approach, discoSnp requires significantly less computational resources, shows similar precision/recall values, and highly ranked predictions are less likely to be false positives. An experimental validation was conducted on an arthropod species (the tick *Ixodes ricinus*) on which *de novo* sequencing was performed. Among the predicted SNPs that were tested, 96% were successfully genotyped and truly exhibited polymorphism.

## INTRODUCTION

Assessing the genetic differences between individuals within a species or between chromosomes of an individual is a fundamental task in many aspects of biology. This is increasingly feasible with next-generation sequencing technologies, as individuals from virtually any species can be sequenced at a modest cost. Of specific interest, single nucleotide polymorphisms (SNPs) are variations of a single base, either between two homologous chromosomes within a single individual, or between two individuals. Finding biallelic or Mendelian SNPs is often done in many biological applications involving SNP genotyping, e.g. population genomics, health, ecology or agronomy research (1,2). To be easily amplified by polymerase chain reaction (PCR), such SNPs must not be surrounded by other polymorphism sources, i.e. other SNPs, indels or structural variants. We call such SNPs *isolated*. Formally, *isolated* SNPs must be distant to the left and to the right by at least *k* nucleotides from any other polymorphism, *k* being one of the main parameters of a SNP detection tool.

State-of-the-art methods to detect SNPs between individuals or strains, generally map sequenced reads (GATK (3), SAMtools (4)) or partial assemblies (DiscoVAR, fermi (5)) on a reference genome. These *reference-based* methods clearly cannot be applied when there is no reference genome. In fact, even when there exists a reference genome, the behavior of these methods is highly dependent on the quality of the assembly. The reality today is that with sequencing costs falling, sequencing efforts are no longer limited to the main species of interest (human and other primates, mouse, rat, drosophila, yeast, *Escherichia coli*, etc.). In fact, biologists are increasingly working on data for which they do not have any close reference genome. Unfortunately, while sequencing with NGS (Next Generation Sequencers) is becoming routine, assembling genomes remains a very complicated task, for which no single software performs consistently well (6), thus producing reference sequences of poor quality. For these reasons, there is a strong need for *reference-free* methods able to detect SNPs, in particular those which are isolated, without relying on a reference genome (reference-free methods).

Reference-free methods that detect SNPs can be broadly divided into two categories. The first category methods perform *de novo* assembly to build a reference sequence. Then, these methods include, as a sub-module, a reference-based method to map back the reads of each individual to this reference sequence (7). We refer to such methods as *hybrid*, as they use both *de novo* assembly and mapping techniques to call SNPs.

A major limitation of these methods is that they tend to cumulate problems raised in assembly and problems raised in mapping. An interesting alternative is to directly focus on the assembly of SNPs, without trying to assemble a full reference. We refer to such methods as *de novo*.

Several methods that fall into the *de novo* category have been developed recently (8–12). These methods are based on a *de Bruijn graph*, i.e. a directed graph where the set of vertices corresponds to the set of words of length *k* (*k*-mers) contained in the reads, and there is an edge between two *k*-mers if they overlap on *k* − 1 nucleotides. KisSnp (8) is a software that takes as input two sets of reads, and detects SNPs using *k*-mers that are differentially abundant between the two datasets. Cortex_var (9) (here after called cortex) detects and genotypes SNPs (as well as indels and larger events), between *n* datasets. bubbleparse (10), which is based on the same graph structure as cortex, detects SNPs that it furthermore classifies into homozygous and heterozygous groups. niks (11) finds homozygous SNPs between two datasets by performing local *de novo* assembly around sample-specific *k*-mers. kSNP v2 (13) proposes yet another approach that is rather dedicated to SNP phylogeny on microbial species. Even if the SNP detection is performed *de-novo*, this method is strongly based on a reference sequence (on which putative selected *k*-mers witnessing a SNP are mapped).

These methods all entail unpractical computational costs (in particular in terms of memory) for large and complex genomes. Even with substantial computational resources, most of them still cannot detect variants in mammalian-size datasets. Therefore, there is still a strong need for robust tools that can detect isolated SNPs without a reference genome.

For these reasons, we introduce a new *de novo* method called discoSnp. This method is designed to call isolated SNPs directly from sequenced reads, without a reference genome. As shown in this paper, discoSnp opens the way to the discovery of SNPs from large-scale studies, even on a standard desktop computer. discoSnp finds and ranks high quality isolated heterozygous or homozygous SNPs from any number of read sets, from 1 to *n*. It introduces new features to distinguish SNPs from sequencing errors or false positives due to approximate repeats. discoSnp can be used for finding high-quality isolated SNPs, either heterozygous, e.g. to build databases of high-quality markers within and across populations, or homozygous between individuals/strains, e.g. to create discriminant markers. The genotyping can be performed as a downstream analysis, based on the read coverage information it provides.

Results presented in this paper show that discoSnp outperforms other reference-free SNP detection methods in terms of resources (faster and using at least two orders of magnitude less memory), type and number of input dataset(s) (ability to find both homozygous and heterozygous SNPs from more than two datasets), and quality of the ranking of predicted isolated SNPs. On two whole-genome mouse datasets, discoSnp detected 2 million isolated SNPs, of which 84% were also found by a manually tuned pipeline in a previous study. On a *S. cerevisiae* dataset discoSnp predicted 90% of the validated SNPs. On an arthropod dataset, discoSnp found 0.3 million isolated SNPs; furthermore, an experimental validation carried over a sample of these SNPs confirmed 96% of them. Finally, discoSnp is designed to reach a wide audience, as it aims to be easy to use, regardless of the size and the complexity of the input data.

## MATERIALS AND METHODS

### Algorithms

**Figure 1.**
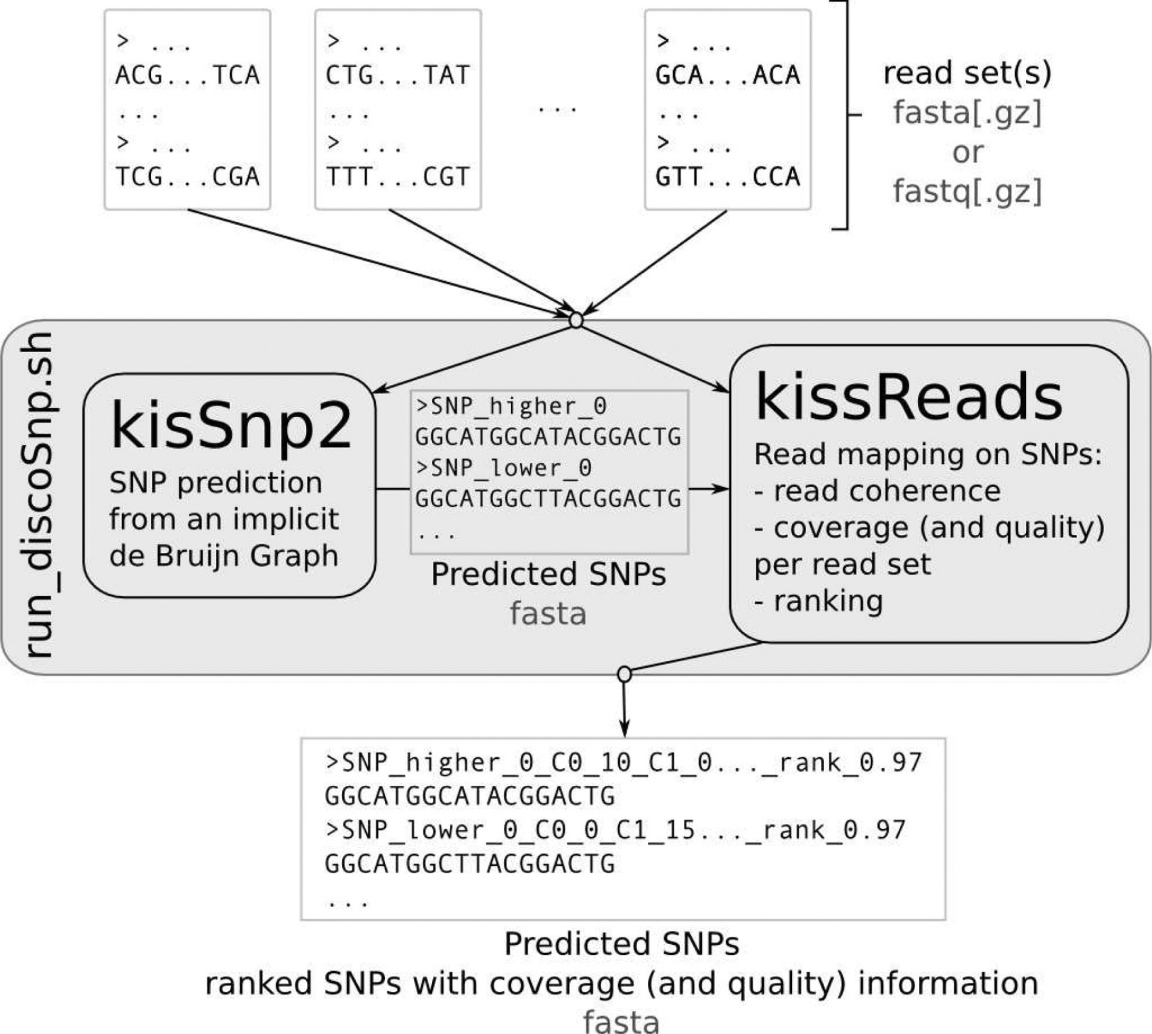
discoSnp method diagram. discoSnp is composed of two modules, KisSnp2 and KissReads that are called by the *run_disco.sh* script.

As depicted in Figure 1 discoSnp is composed of two modules, KisSnp2 and KissReads. KisSnp2 detects putative SNPs from one or more sets of reads. KissReads evaluates the coverage and base quality of the SNPs per read set and ranks them accordingly. These two modules are independent, however, a script automatically pipelines them, so their calls are transparent to the user.

#### KisSnp2 module

**Figure 2.**
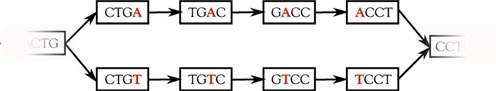
Toy example of a *bubble* in the *de Bruijn Graph* (*k* = 4). Bubble generated by a single nucleotide polymorphism. The two polymorphic sequences are …*CTGACCT*… and …*CTGTCCT*…

Similarly to cortex and KisSnp, the KisSnp2 module is based on a *de Bruijn graph*. A *de Bruijn graph* is a directed graph that contains all the *k*-mers present in the read dataset as vertices, and all the possible (*k* − 1)-overlaps between *k*-mers as edges. Such graphs have been widely used in *de novo* assembly (14). The idea is the following: if a dataset contains two sequences that are identical, except for one character, then these sequences generate a *bubble* in the graph (see an example in Figure 2). Formally, a *bubble* in a *de Bruijn graph* is composed of two distinct paths of *k* + 2 nodes, having the start and the end nodes in common. Precisely, KisSnp2 detects couples of paths of length 2*k* − 1, denoted by *p*_1_ and *p*_2_, that correspond to polymorphic sequences, i.e. sequences of bubbles excepting the two extreme nodes. Formally, these two paths can be written as *p*_1_ = *p*α*q* and *p*_2_ = *p*β*q*, with *p, q* being (*k* − 1)-mers, and α and β are single nucleotides such that α ≠ β.

In KisSnp2, this idea is exploited by generating the *de Bruijn graph* of all input dataset(s) pooled together, and by searching the previously described bubbles in the graph. To avoid *k*-mers generated by sequencing errors, only *k*-mers whose support (coverage) is above or equal to a user-defined threshold, *c*, are taken into account in the construction of the *de Bruijn graph*. The *de Bruijn graph* is built with the minia data structure (15,16). The nodes of the graph are stored in a cascade of Bloom filters. As the edges of the graph can be inferred from the nodes, they do not need to be stored. Overall, the graph representation requires around 1 byte per *k*-mer. This data structure supports efficient and exact enumeration of the neighbors of any node in the graph. Thus, it enables to efficiently traverse the *de Bruijn graph* starting from any node, on both forward and reverse strands.

In the following, we say that a *k*-mer ω can be *right extended* with a nucleotide α if the *k*-mer obtained by concatenating the suffix of length *k* − 1 of ω with α, exists in the data structure. Symmetrically, we say that a *k*-mer ω can be *left extended* with a nucleotide α, if the concatenation of α with the prefix of length *k* − 1 of ω forms a *k*-mer that can be found in data structure.

The KisSnp2 algorithm detects all *k*-mers that can be right extended with at least two distinct nucleotides. Such *k*-mers are the starting nodes of the bubbles they generate, as in the example depicted in Figure 2, where the *k*-mers obtained after the extension are *CTGA* and *CTGT*. Then, for each such couple of *k*-mers starting with the same *k* − 1 length prefix, KisSnp2 tries to complete the bubble by performing successively *k* − 1 right extensions on both paths with the same nucleotide (using successively nucleotides *C, C* and *T* for the example in Figure 2). If, at one step, both paths cannot be right extended with the same nucleotide, then the bubble is discarded. Based on this pattern, only *isolated* SNPs can be detected, thus avoiding SNPs that are closer than *k* bases to other polymorphisms.

##### Branching bubbles

High copy number repeats in genomes typically yield complex bubbles, which may combinatorially increase the number of false positives. To limit this effect, a classic filter consists in removing bubbles that contain at least one branching node; this is the default behavior of discoSnp (−b 0). The counterpart of this filter is that it may discard true bubbles that contain branchings only due to repeats or to some isolated non-filtered sequencing errors. To achieve a better compromise between false positive and false negative rates, especially in complex genomes, we introduce a novel concept of symmetrically branching bubbles.

**Figure 3.**
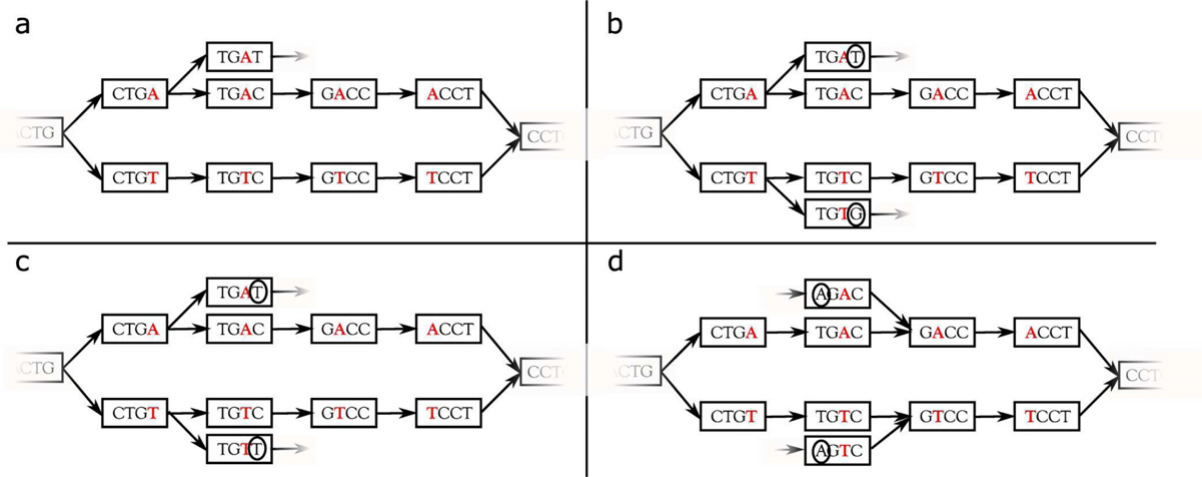
Examples of non-symmetrically branching bubbles (**a** and **b**) and symmetrically branching bubbles (**c** and **d**). Path divergences in bubbles a and b create branching bubbles, but the branching is not symmetric. Divergence is only present in one path (**a**) or in both paths but with distinct (circled) characters (**b**). Path divergences in bubbles c and d create symmetrically branching bubbles. Both paths of these bubbles can be right extended (**c**) or left extended (**d**) with the same two (circled) characters. With option *−*b 0 (default), none of these bubbles would be considered as a SNP, with option −b 1 bubbles **a** and **b** would have been considered as SNP while with option −b 2 all of them would have been output.

More precisely, a bubble is called *non-branching* if during its construction, for every extension step, there is only one *k*-mer that can be used (only one possible extension for each of the two paths). On the other hand, if at some point during the extension of the bubble, there is a choice between two *k*-mers (for at least one of the paths), the bubble is said to be *branching*. Now, if at some point during the extension, both paths can be extended with the same (at least) two nucleotides, then the bubble is called *symmetrically branching* (see examples in Figure 3). Finally, bubbles that are branching, but not symmetrically branching, are called *simply branching*. Note that branchings are checked in both directions (left and right).

The rationale for keeping simply branching bubbles is that they may correspond to true SNPs, where the branching is simply due to a sequencing error that accidentally passed the coverage filter. As shown in the ‘Results’ section, this enables to increase the recall especially in complex genomes, with high copy number repeats, while being highly efficient in removing the numerous false positive branching bubbles. discoSnp can be run with any of these three filtering strategies: filtering out all branching bubbles (default behavior, option −b 0), or filtering out symmetrically branching bubbles only (−b 1), or no filtering based on the branching criteria (−b 2).

##### Retrieving sequence contexts

Each sequence in the multi-fasta file output by KisSnp2 is composed of the 2*k* − 1 nucleotides, which correspond to the bubble, together with its left and right contigs that are extending the 2*k* − 1 sequences. For every such contig, the length of the longest unambiguous context (longest non-branching path in the graph starting from the bubble) is also indicated. Reconstructing these contexts is done with the Minia assembler (15) algorithm. Its contiging algorithm was evaluated within the Assemblathon2 challenge (17) (team ‘*Symbiose*’).

#### KissReads module

One should note that it is not possible to compute read coverages, nor read quality information, based solely on a raw *de Bruijn graph*. Moreover, all *de Bruijn graph*-based methods may build chimeric sequences due to overlapping *k*-mers, which are never present in the same read. These sequences are said to be *non-read-coherent*. The KissReads module is meant to filter out bubbles composed of *non-read-coherent* sequences, to add coverage and quality information on the remaining ones, and finally to rank SNPs (see the section below).

Given a sequence *s* and a set of reads $\mathcal {R}$ mapped on *s*, we say that *s* is *read-coherent* if, for each position of *s*, at least *c* reads are mapped, with *c* a user-defined threshold. Note that reads are mapped in a semi-global manner: mapped reads may have a prefix starting before the first position of *s* and/or may have a suffix ending after the last position of *s*. By default, one substitution is authorized which roughly corresponds to current error rates. Moreover, knowing that in the discoSnp framework the sequence *s* represents one of the two alleles of a SNP, no substitution is authorized on the polymorphic position during the mapping.

**Figure 4.**
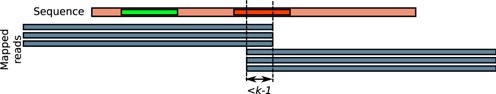
Read-coherency and *k*-read-coherency example. With coverage threshold = 2, schematic example where a sequence is *read-coherent* but not *k-read-coherent*. The leftmost represented *k*-mer (green) on the sequence is an example where the *k*-mer starting at this position is covered with three mapped reads. On the other hand, the rightmost represented *k*-mer (red) is covered by no read, thus illustrating why the sequence is not *k-read-coherent*.

Moreover, it may appear that a position of *s* is mapped only by the *end* of reads (last *k* positions), and/or only by the *beginning* of reads (first *k* positions). This reveals a situation where part of the sequence *s* was generated by *k*-mers not truly belonging to the mapped reads, i.e. due to a repeat of length bigger or equal to 2*k* and smaller than the read size. Such a sequence is thus chimeric. An example of this situation is illustrated in Figure 4. To overcome this problem, we define the *k-read-coherency*. Given a sequence *s* and a set of reads $\mathcal {R}$ that can be mapped on *s*, we consider *s* as *k-read-coherent* if, for each position *i* of *s* except the last *k* − 1 ones, there exists at least *c* reads that fully map on the *k*-mer starting on position *i*. Note that this condition is symmetrical, whether *s* is read on both forward or reverse complement strand.

Given the set $\mathcal {S}$ of pairs of 2*k* − 1 sequences generated by KisSnp2 and the initial sets of reads, the KissReads algorithm maps the reads (using a classic seed-and-extend approach) on the sequences of $\mathcal {S}$. Once all reads are mapped on all sequences of $\mathcal {S}$, the *k-read-coherency* is computed for each read set and for each sequence of $\mathcal {S}$. Sequences of $\mathcal {S}$ for which at least one read set makes them *k-read-coherent*, are conserved. KissReads outputs such coherent sequences together with their read depth per read set and with the average PHRED quality of the polymorphic nucleotide per read set.

### Ranking SNPs

discoSnp scores each SNP according to the coverage repartition of its alternative paths between the conditions. The aim is to rank best the SNPs that are the most discriminant between the samples. For a given SNP, the score is the *Phi coefficient* of the table of read counts for each path and each dataset, computed as follows: $\sqrt{\frac{\chi ^2}{n}}$. It can be seen as a normalized Chi-squared statistics that varies between 0 and 1. A high score, close to 1, is obtained if the frequencies of the paths are very different between datasets, the best case being for homozygous SNPs between two datasets, where each path is strictly specific to one dataset. Notably, this score ranks poorly bubbles that are due to sequencing errors or inexact repeats as they are likely to have similar repartitions in all datasets (small frequency for an error, and equal frequency for repeats). Moreover, the normalization prevents from over-scoring highly covered bubbles which are often due to repeats. Since this ranking favors SNPs for which one variant is enriched in one read set, it is not well suited to select SNPs that are heterozygous in all read sets. It is also not usable with only one dataset, one diploid individual or a pooled sample. As shown in the ‘Results’ section, other rankings can be used in these cases.

### discoSnp input and output

In summary, discoSnp takes as input any number of read sets, and has two main parameters *k* and *c*, respectively the *k*-mer size used to build the *de Bruijn graph* and the minimal coverage a *k*-mer should reach to be inserted in the graph. When applied to two read datasets or more, discoSnp is executed only once as all datasets are pooled together. All isolated SNPs shared by any number of samples are output as single calls.

Finally, the discoSnp output is a sorted *multi-fasta* file, in which every consecutive couple of sequences corresponds to the two 2*k* − 1 paths of a SNP, surrounded by its left and right contigs. Headers of the sequences give information about read coverage and average PHRED quality for each input dataset, lengths of unambiguous left and right extensions, and Phi coefficient of each SNP.

### Simulated datasets and evaluation

We propose here an overview of the evaluation approach. Supplementary details on the protocols for generating data and for evaluating the results are provided in Supplementary Additional File 1.

All tools were run with at least *k* = 31 and a minimal coverage of 4 (*k*-mers that were seen less than four times were considered to be sequencing errors) or based on the developers’ advices. When several parameter sets were tested, presented results were obtained with those giving the best results. Full details on parameters and full results are proposed in the Supplementary Additional File 1.

#### Simulation and evaluation for one or two diploids

In order to evaluate the behavior of discoSnp on large, complex genome sequences, and to compare it to the other reference-free SNP discovery tools, we propose an experiment simulating the sequencing of two diploid, human chromosome 1 individuals.

To obtain a realistic distribution of SNPs and genotypes, we used SNPs predicted from real human individuals. Two vcf files were retrieved from the ‘1000 genome project’ (phase 1 release), corresponding to the human chromosome 1 of two individuals: HG00096 and HG00100. We then generated the genome sequences for the two diploids, i.e. two sequences per individual, by placing the substitutions listed in the vcf files on the human reference sequence (GRCh37/hg19 reference assembly version). In the case of a homozygous SNP, the same nucleotide was placed on the two sequences, while for a heterozygous SNP, one sequence was randomly chosen for each of the two nucleotides.

A total of 316 502 positions were mutated, with 131 263 positions that were mutated in the same time in both individuals (representing an average ratio of 0.5 SNPs by kb in each individual). 29 038 SNPs (9%) have a homozygous genotype in both individuals (homozygous-homozygous), 218 556 (69%) are heterozygous in only one individual (homozygous–heterozygous) and the remaining 68 908 (22%) are heterozygous in both individuals.

We then simulated a 40x coverage sequencing of these two individuals, with 100-bp reads and 1% error rate, thus generating 89 753 907 reads. The sequencing procedure is further explained in Supplementary Additional File 1.

Among the simulated SNPs, only those distant by more than *k* nucleotides from one another were selected (isolated SNPs) and were recorded as 2*k* − 1 bubbles in a reference file (same format as the output of discoSnp). This gave 150 348 heterozygous isolated SNPs for individual HG00096 (84% of all heterozygous mutations inserted in this individual) and 260 539 isolated SNPs when considering both individuals (82% of all simulated SNPs). We produced two SNP reference files, $ref\_snps.fa$ (as described in ‘Precision and recall computation’, in Supplementary Additional File 1): one for the HG00096 individual, and a second one when considering the two individuals. Predicted SNPs were compared to one of these files (depending on the dataset(s) on which discoSnp was applied) in order to compute the number of true and false positives. A predicted SNP for which the two paths match exactly the two paths of a simulated SNP present in this reference file is a true positive (TP), else it is a false positive (FP). In the same way, a SNP from the reference file not matched by any predicted SNP is a false negative (FN). The recall is given by the number of TP divided by the number of TP plus the number of FN. The precision is given by the number of TP divided by the number of TP plus the number of FP. When allowing branching bubbles (option −b 1), precision was computed by considering as false positives only bubbles that do not correspond to any simulated SNP (isolated or not). Full details on the evaluation protocol can be found in the Supplementary Additional File 1. Isolated SNPs detected with other tools than discoSnp were transformed into the discoSnp output format, in order to be evaluated with the same protocol.

#### Simulation and evaluation for two and more haploids

We propose an experiment simulating more than two haploid bacterial individuals. For doing this, we created 30 copies (that we call *individuals*) of the *E. coli* K-12 MG1655 strain. We then simulated SNPs with a uniform distribution such that ≈4200 SNPs (≈0.1% of the genome length) are common to any pair of individuals, half this number is common to any trio of individuals, a third to any quadruplet, and so on. With this strategy, while considering all the 30 individuals together, 69 600 SNP sites were generated, covering ≈1.5% of the genome. Similarly to the diploid dataset, we simulated a 40x sequencing of each of the 30 individuals, with 100-bp reads and 0.1% error rate. Thus, 1 855 870 reads were generated per read set.

Finally, we created a reference file containing the isolated-SNPs per subsets of individuals: the first two, the first three, and so on. For each of these subsets, a SNP was considered as isolated if no other SNPs were simulated in the *k* − 1 positions before and after the SNP's locus, inside this subset. In the presented results, for two individuals, 4164 simulated SNPs are isolated while 268 SNPs (≈6%) are not. For 30 individuals, 25 993 SNPs are isolated while 333 930 (≈92%) are not. For the isolated SNPs, the two corresponding sequences of length 2*k* − 1 were used as a reference to assess the precision/recall of all tested tools, based on the same protocol as the one used for two diploids.

### Mouse dataset

discoSnp was applied on a real dataset to compare two mouse inbred strains, *FVB/NJ* and *C57BL/6NJ*, the latter corresponding to the mouse reference genome (NCBIM37). Reads were retrieved from the European Nucleotide Archive (www.ebi.ac.uk/ena/), 987 million reads for the *C57BL/6NJ* strain (ERP000041) and 1888 million reads for the *FVB/NJ* strain (ERP000687).

To compare with discoSnp results, we retrieved the set of predicted SNPs obtained with the same data by Wong *et al.* (18) (www.sanger.ac.uk/resources/mouse/genomes/, file mgp.v3.snps.rsIDdbSNPv137.vcf). We selected SNPs having distinct genotypes in *FVB/NJ* and *C57BL/6NJ* strains. This provided 5 020 489 SNPs, among which 2 472 456 (≈49.2%) are isolated. We evaluated the number of isolated SNPs found by the two methods and those found specifically by one method or the other, by comparing the discoSnp output to the Wong *et al.* one. The set of SNPs that was predicted by Wong *et al.* was transformed in discoSnp format, as for the other experiments, in order to allow the comparisons.

### *Saccharomyces cerevisiae* dataset

discoSnp was applied on a real *S. cerevisiae* dataset, for which several SNPs have been biologically validated in a recent study (19). In this study, three glucose-limited, chemostat-evolved populations of haploid S288c, named E1, E2 and E3, were sequenced every ≈70 generations, giving eight samples per population. Using a reference-based mapping approach, 110 mutations were discovered, among which only 33 have a minor allele frequency (MAF) >10% and were confirmed by Sanger sequencing. Among these 33 SNPs, 30 are isolated (with *k* = 31). Therefore, in order to assess the performance of discoSnp on this dataset in terms of recall, these 30 SNPs were used as a reference. The 24 read sets were downloaded from the NCBI Sequence Read Archive (with the accession number SRA054922), and processed to remove barcode and adapter sequences as in the initial study.

discoSnp was run independently on populations E1, E2 and E3. For each population, discoSnp was applied on the eight read sets corresponding to the eight time points, with the default parameters and *c* = 11.

Details about datasets and applied commands are provided in Supplementary Additional File 1.

### Tick dataset

discoSnp was applied on real sets of reads as part of a population genetic study of the tick *Ixodes ricinus* (2). DNA was extracted from two tick pools, one composed of ten individuals from Gardouch (close to Toulouse), France, and another composed of 20 individuals from Malville (close to Nantes), France. A genomic reduction was applied on these two pools by selecting the piece of an agarose gel containing DNA fragments, with a length of 500–600 nucleotides within the smear obtained following the enzymatic digestion by Msel of genomic DNA. This reduction corresponded to 3.8% of the initial genome. The DNA was sequenced by 454 Roche pyrosequencing, leading to the generation of 1 389 201 reads in two libraries (730 482 for one and 658 719 for the second). After quality trimming (where reads or ends of reads with a PHRED quality score inferior to 20 or that did not contain the expected restriction site were deleted), a total of 996 508 reads (536 061 for the first pool and 460 447 for the second) with an average length of 529 bp were used for analysis with discoSnp.

In order to be able to design efficient primers, detected SNPs were then selected for experimental validation using the following criteria : (i) SNPs with a coverage between 4 and 10 (126 567 SNPs) were selected to avoid sequencing errors and repeated sequences, highly frequent in ticks (66% of the genome is repeated (20)), (ii) SNPs had to be distant from homopolymers (the bubble sequences should not contain any window of 8 nucleotides with at least six identical nucleotides), and they must not be closely located (less than *k* bp) to any other variants such as indels or other SNPs (reads were mapped to the bubble sequences using gassst (21) with a similarity threshold set at 80%, bubbles with at least one read mapped with differences were excluded) and (iii) SNPs with PHRED sequence quality <30 were filtered out. As in this study framework one is not interested by SNPs discriminating the two datasets, we did not use the Phi coefficient for selecting SNPs for experimental validation. Among the 1768 SNPs meeting these criteria, 384 were randomly picked for genotyping validation.

Primers were designed for each selected SNP. To validate them, we performed a genotyping run using *Fluidigm* technology, where primers are combined with fluorochrome (VIC or FAM for each allele) (22). Reading the fluorescence allows to determine the genotype of the individual typed at each locus (homozygous XX or YY, heterozygous XY).

## RESULTS

We propose experiments that aim at (i) assessing the quality of discoSnp results on simulated datasets, in comparison with state-of-the-art reference-free SNP detection methods (including a *hybrid* approach); (ii) showing how discoSnp performs on real data, with biological validation. In addition, the computational resources (time and memory) required by discoSnp are compared with those required by the other tools.

Results were obtained with discoSnp, version 1.2.1 available online, as well as with the latest versions of NIKS, bubbleparse (released on April 2013) and cortex (Cortex_var v1.0.5.21). The tests were performed on the *GenOuest* (genouest.org) cluster, composed by Intel Xeon^©^ core processors with speed varying between 2 and 2.8 GHz.

### Detection of isolated SNPs from two to 30 haploid datasets simulated from *E. coli*

We simulated 30 read sets from *E. coli* genomes, in which SNPs were inserted in order to mimic a realistic allele frequency spectrum of several individuals (see ‘Materials and Methods’ section for details). Below, we present the results obtained by different tools on (i) two haploids, and on (ii) three to 30 haploids, with respect to the set of isolated SNPs.

#### Results on two haploids

Results of discoSnp applied with default parameters on 2 of the 30 haploid genomes serve as proof of concept of the approach. Results reach high precision and recall: 98.81% of predicted SNPs are true positives, while 97.31% of simulated isolated SNPs were recovered. Moreover, these results were obtained in 3 min 49 s, and needed no more than 7MB of RAM memory. Results presented in Table 1 show that, on a simple dataset composed by two bacterial genomes, all tools give similar results in terms of precision/recall (except for kissnp and niks, all results have precision ≈99.19 ± 0.66% and recall ≈96.56 ± 1.41%). However, discoSnp runs faster than other tools while being by far the one needing the smallest amount of memory, by at least three orders of magnitude.

**Table 1.** Comparative results of several tools discovering SNPs between two haploid bacterial datasets

Tool	Precision	Recall	Time (s)	Memory (GB)
kissnp	98.03	90.58	1234	50.6
niks	77.50	72.11	67217	86
bubbleparse	98.53	97.98	980	12.6
cortex	99.85	95.15	289	9.5
discoSnp	98.81	97.31	229	0.007
*hybrid* assembly	97.79	98.36	430	6.3

#### Results on more than two haploids

**Figure 5.**
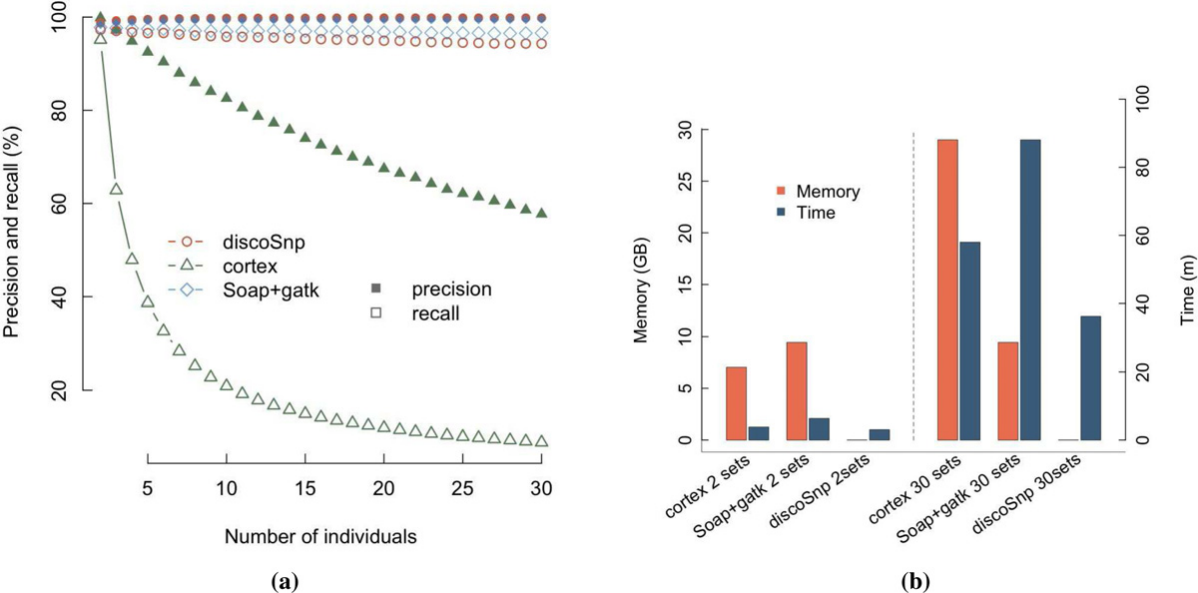
discoSnp, cortex and hybrid strategy (soap + gatk) results, depending on the number of input haploid individuals. Soap and gatk were launched with default parameters. For discoSnp and cortex, *k*-mers having three or fewer occurrences in all datasets were removed. (**a**) Precision and recall: filled symbols represent the precision and empty symbols represent the recall. (**b**) Time and memory performances for two (left part) and 30 (right part) individuals.

When applied on more than two datasets, discoSnp still produces high quality results, with precision reaching 99.83%, and recall decreasing only slightly (94.29%) for 30 individuals (Figure 5a). Notably this shows that discoSnp results remain constant regardless of the number of input datasets, unlike cortex whose recall and precision drop for more than two datasets. Except cortex, none of the other *de novo* tools could be run on more than two datasets. As shown in Figure 5b, discoSnp runs faster than cortex, while needing much less memory: applied on 30 individuals, cortex requires 3 h 38 s and 9658MB of memory while discoSnp runs in 37 min and requires 9MB. For all methods, running time grows linearly with respect to the number of read sets, but with a smaller coefficient for discoSnp (see Supplementary Figure S3 of Supplementary Additional File 1).

Furthermore, in Figure 5a and in b, we present the results obtained by the hybrid approach when assembling one of the 30 read sets for creating a reference sequence (with SOAPdenovo2 (23)) and then mapping the read sets to this reference (with Bowtie 2 (24)). Finally, SNPs were called with GATK (25). The results are similar to discoSnp ones, with a slightly better recall and a slightly worse precision. In this particular case, for which the reference dataset is small, complete, well covered and easy to assemble, a hybrid method can be preferred to the *de novo* ones, as long as the time and the memory footprints are not limiting.

### Detection of isolated SNPs from diploids, simulated from human chromosome 1

In this section, we present various results obtained by discoSnp on a more complex genome, namely diploid read datasets simulated from two human chromosome 1 individuals, to discover both heterozygous and homozygous SNPs. As expected, when the complexity and the repeat content of the input genome increase, discoSnp makes more erroneous calls and misses more real SNPs. Whereas precision remains reasonable, with <3% of false positive calls, recall is more impacted with 72% of the isolated SNPs that are recovered.

Among the 72 518 SNPs that are missed, 86% fall in repeated regions of human chromosome 1 (as masked by RepeatMasker from UCSC Genome Browser). In order to increase the recall and detect some of the SNPs located in repeated regions, we can change the filtering parameter that controls the branching features of the detected bubbles. By default, only clean bubbles without any branching are kept. Allowing all kinds of branching bubbles (option −b 2) would lead to recover almost all simulated SNPs but at a cost of millions of false positive calls and a precision close to zero. As a compromise, when allowing only some of the branching bubbles, those without any symmetrical branchings (option −b 1), recall reaches 79.22%, allowing to detect some of the SNPs that were simulated inside repeated regions of the chromosome. This shows that symmetrically branching bubbles are the most common type of branching bubbles and are mainly false positives. Interestingly, among the few symmetrically branching bubbles that correspond to real SNPs, 90% are located in highly repeated regions such as transposable elements, and specifically SINE elements (59%).

**Figure 6.**
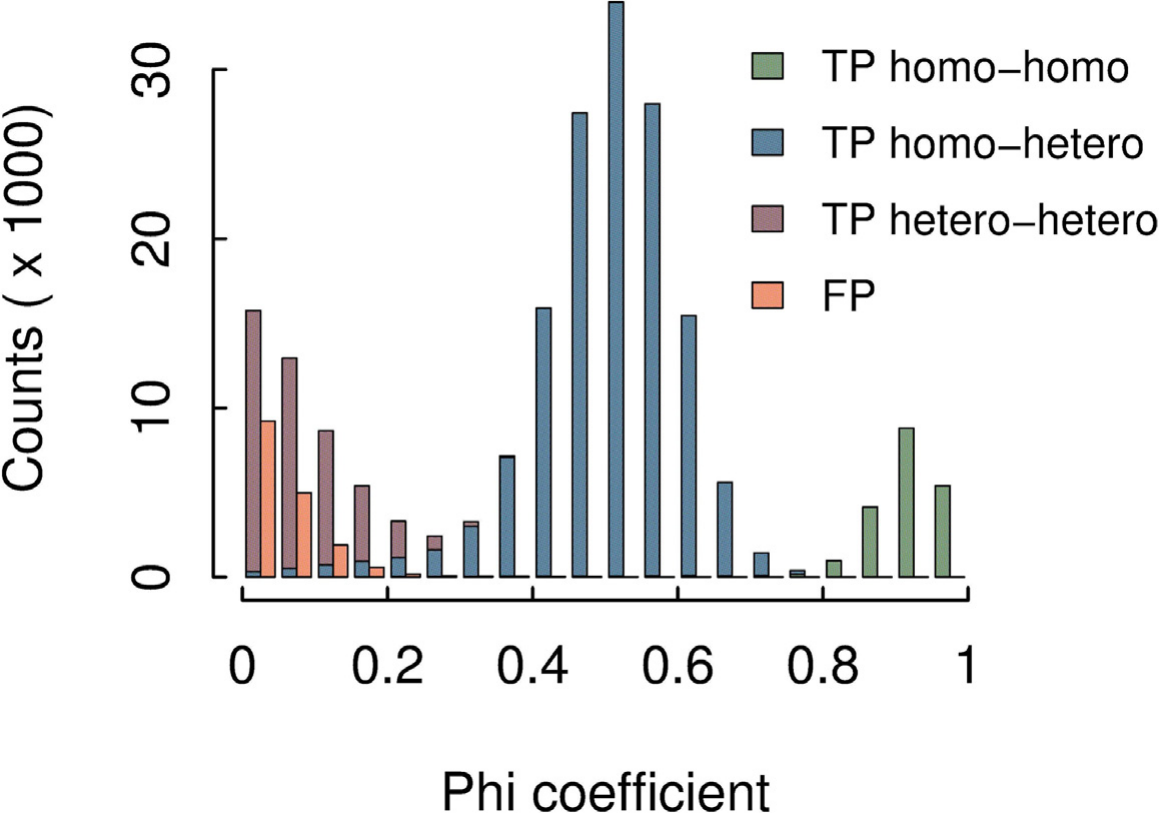
Repartition of SNPs detected by discoSnp depending of their phi coefficient. FP are false positives and TP are true positives. Homo (resp. hetero) stands for homozygous (resp. heterozygous) SNPs. True positive SNPs are then classified according to their genotype in the two individuals.

The precision obtained when using the −b 1 parameter is slightly lower, 92.33%, with 17 153 false positives. We observed that a large majority (78.8%) of these false calls are inexact repeats of size at least 2*k* − 1 contained in the chromosome 1, with both paths of the bubble mapping exactly to the non-mutated chromosome. Consequently, these bubbles would appear in any individual and could be easily filtered out when comparing coverage values between individuals. This is, in fact, the purpose of the phi coefficient, to rank the predictions according to how discriminant their coverages are between individuals, leaving almost all false positives with the lowest ranks. As shown in Figure 6, 99.7% of SNPs ranked with a phi value >0.2 are real SNPs. This filter will remove however most of the SNPs that are heterozygous in both individuals, but for SNPs that are discriminant between the individuals, i.e. for which at least one individual is homozygous, the recall stays high (above 77.8 % over such SNPs).

If the user is interested specifically in non discriminant polymorphisms, or in the case when only one individual is analyzed, we recommend to use an alternative filter based on the left and right unambiguous extension lengths (see ‘Materials and Methods’). Long unambiguous extensions reveal SNPs that are isolated from other polymorphisms, while short ones reveal repeated regions or regions with high densities of polymorphism. Consequently, false positives have smaller unambiguous extension sizes (median size of 24 bp versus 527 bp for true positives).

In addition to state-of-the-art *de novo* SNP detection tools (cortex and bubbleparse), we compared discoSnp to a *hybrid* strategy consisting of three steps: *de novo* assembly using SOAPdenovo2 (23), mapping with Bowtie 2 (24) and SNP calling using GATK (25). Qualitatively, the precision and recall values remain comparable between all tested methods on this dataset. Table 2 shows that, with *stringent* parameters (*d* = 0 for bubbleparse and *b* = 0 for discoSnp), all tools have a precision of 96.50 ± 0.72% and a recall of 71.20 ± 1.50%. With more sensitive parameters (*d* = 1 for bubbleparse and *b* = 1 for discoSnp), discoSnp achieves the best precision/recall compromise, notably with the highest recall value (79.22%).

**Table 2.** Results obtained by several tools with several parameter configurations on two human chromosome 1 diploid datasets

Tool	Precision (%)	Recall (%)	Time	Memory (GB)
discoSnp*b*0	96.98	71.99	**4h24**	**5**
cortex	**97.22**	69.70	10h09	105
bubbleparse*d*0	95.78	72.71	11h24	105
discoSnp*b*1	92.33	**79.22**	4h44	**5**
bubbleparse*d*1	91.66	76.60	11h00	105
*hybrid* strategy	96.18	72.86	10h34	54

The upper part of the table shows results obtained by *de novo* tools with stringent parameters (no branching allowed in bubbles). Below are the results obtained with the same tools but with more sensitive parameters, namely allowing for some branchings in bubbles. The bottom line shows results obtained with a hybrid strategy (SOAPdenovo2 + Bowtie2 + GATK), filtering out low covered SNPs (with the same parameters as those applied for all the other tools, by removing SNPs whose both alleles have coverage below four). As presented in Supplementary Additional File 1, results are worse without this filter.

**Figure 7.**
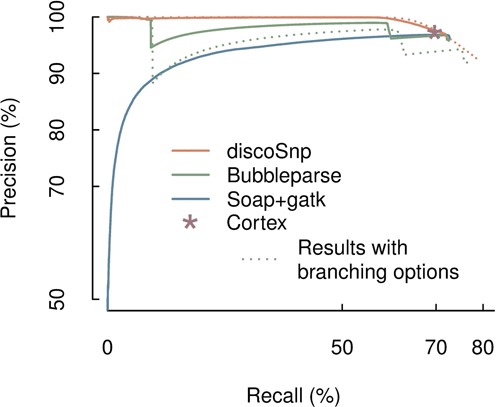
Comparative results of discoSnp, cortex, bubbleparse and the *hybrid* SOAPdenovo2 + Bowtie2 + GATK approaches on the two diploid human chromosome 1 dataset. Precision versus recall curves are obtained by ranking the predicted SNPs. Each data point is obtained at a given rank threshold, where precision and recall values are computed for all SNPs with better ranks than this threshold. In this framework cortex does not rank the predicted SNPs, its results are thus represented by a single point. Plain lines for discoSnp and bubbleparse were obtained while discarding all branching bubbles (options −b 0 and depth = 0 respectively), whereas dotted lines were obtained when allowing for some branchings (options −b 1 and depth = 1 respectively).

Figure 7 shows a precision–recall curve for each software, according to its own ranking method. Unlike discoSnp and Bubbleparse, the hybrid approach gives high ranks to many false positive predictions. We therefore conclude that ranks are not a viable indicator to filter out the false positives for this method. The discoSnp ranking approach enables to reach a precision of ∼100% for a high (≈60%) recall range, slightly outperforming Bubbleparse.

**Figure 8.**
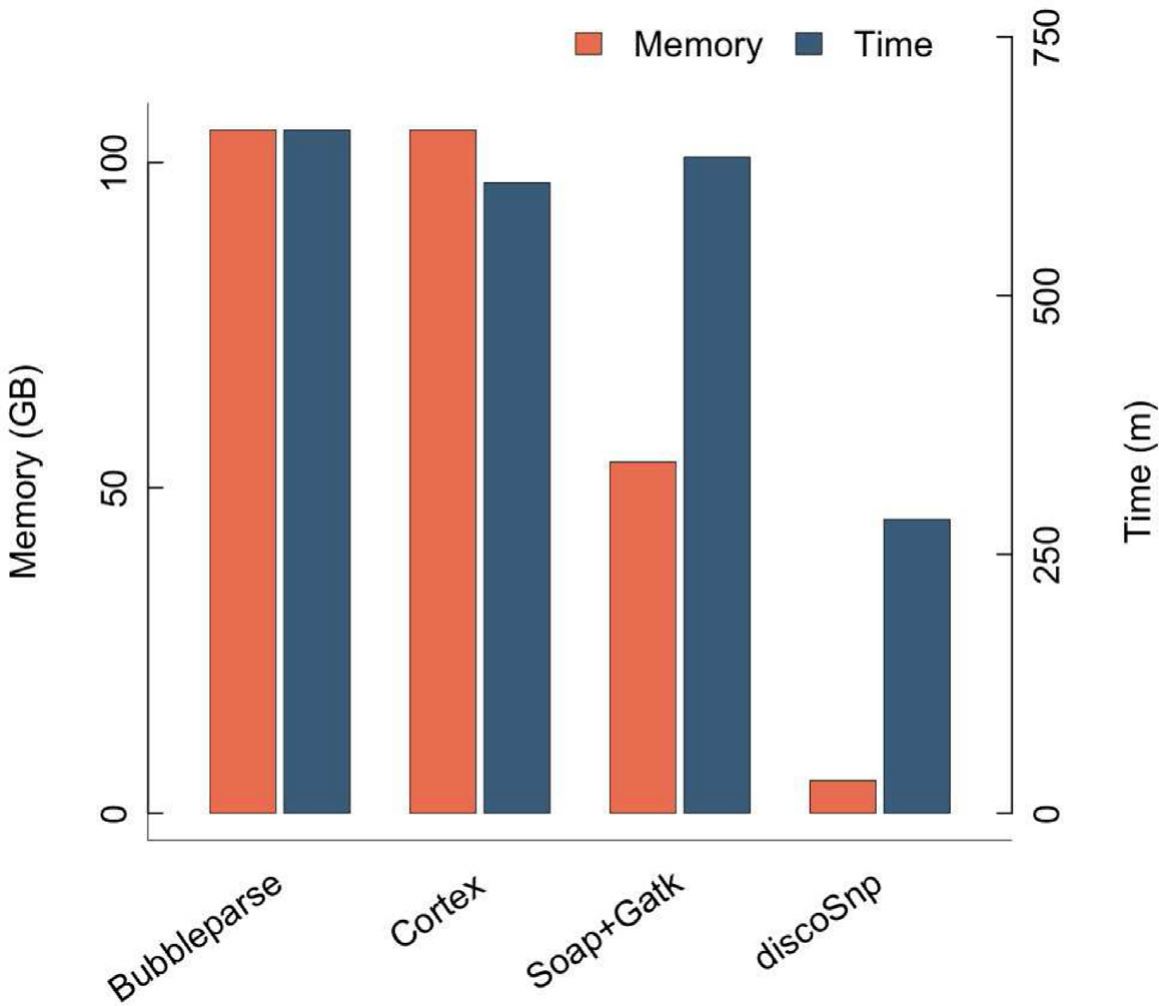
Comparative memory and time performances on the human chromosome 1 dataset. Time values are given with options *depth* = 1 for bubbleparse and −*b* 1 for discoSnp.

As shown in Figure 8, an important benefit of discoSnp stands in the computational resources that are needed. discoSnp is at least twice faster than any other compared tool, while its memory needs are lower by several orders of magnitude than all other approaches.

Finally, it can be of interest to detect SNPs from one unique set of reads representing an individual or a pool of individuals, as this is the case, for instance, in the tick study presented below. Therefore, we performed similar tests while searching for heterozygous SNPs from one simulated set of reads sequenced from a single diploid individual. Conclusions of this study are similar to previous ones as, with the default and most stringent filtering parameters, 67% of isolated SNPs are recovered, with roughly the same amount of false positive calls (precision of 94.1%).

### Results on real data: detection of SNPs from two mouse strains

To analyze the behavior of discoSnp on real data, we detected SNPs between two publicly available mouse datasets produced by an Illumina GA2 sequencer. Taken together, these datasets contain 2.88 billion of 100-bp reads. The first one was generated from the *FVB/NJ* mouse inbred strain, while the second one was generated from the *C57BL/6NJ* reference line. A previous study by Wong *et al.* (18) mapped the reads from the *FVB/NJ* strain to the *C57BL/6J* reference sequence, and detected ≈5 million homozygous SNPs, among which 2 472 456 are *isolated* SNPs. In the following, we refer to their set of isolated SNPs as *IS*.

**Figure 9.**
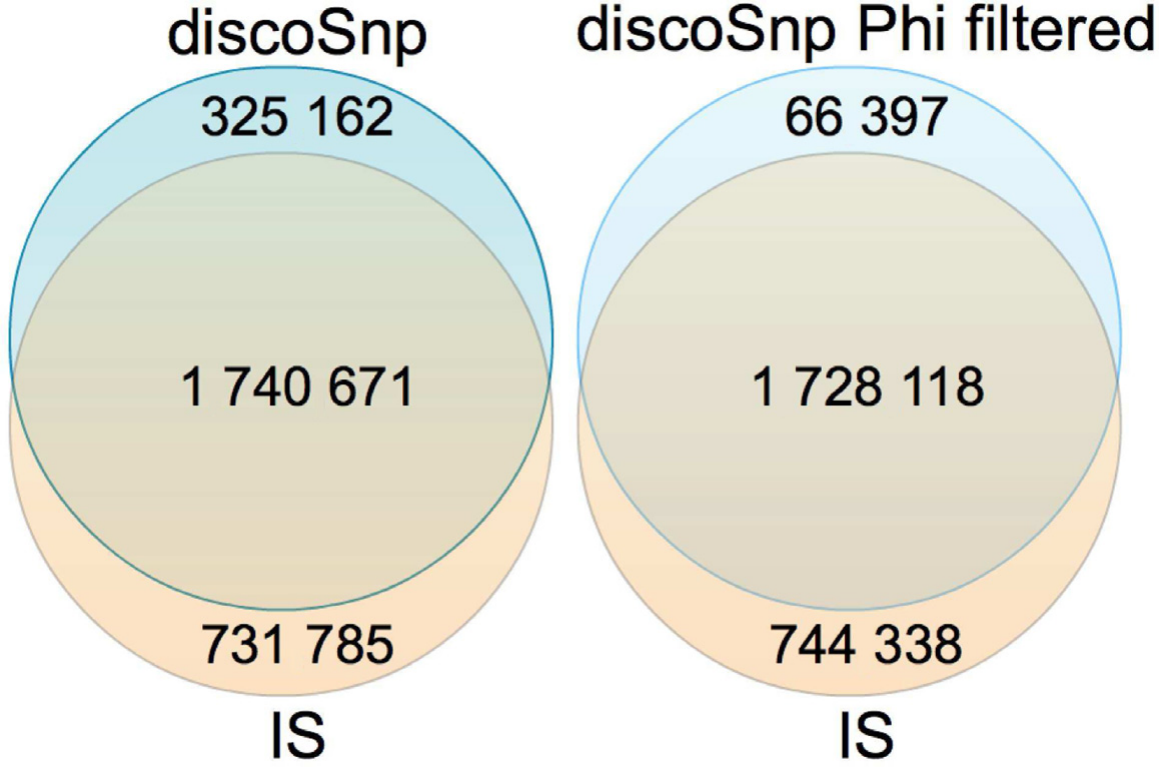
Venn diagrams of isolated SNPs detected by Wong *et al.* (18) (*IS* set) and by discoSnp. **Left**: Raw discoSnp results. **Right**: Filtered discoSnp results, i.e. SNPs with Phi ≥0.2.

discoSnp was run with *k* = 31 and *c* = 5 on the *FVB/NJ* and *C57BL/6NJ* read sets, and discovered 2 065 833 isolated SNPs. As presented in Figure 9 (left), 84.3% of these SNPs are also present in *IS*, while 70.1% of the *IS* set is detected by discoSnp. As the study was performed on inbred strains, only homozygous SNPs are expected. Therefore, we can take advantage of the Phi coefficient to further filter discoSnp predictions. By removing SNPs for which the Phi coefficient is ≤0.2, the number of predicted SNPs drops to 1 794 515. As depicted in Figure 9 (right), 96.3% of the filtered SNPs are also in *IS*, while roughly the same proportion of *IS* SNPs are found, compared to the unfiltered results (70.0%). Therefore, by keeping only SNPs with a Phi coefficient greater than 0.2, we discard almost exclusively SNPs that are found only by discoSnp, and which might be false positives.

Note that in this experiment, we can not use the terms ‘precision’ and ‘recall’ for describing results quality. Indeed, the *IS* dataset was obtained via a mapping approach, and thus it can neither be considered as an exhaustive, nor a perfect list of isolated SNPs.

It is worth stressing that the results of Wong *et al.* were obtained by running a complex pipeline, involving a high-quality reference genome, 6 distinct tools, followed by a filtering step composed of 14 non-automated filters (coverage, quality, genotype, etc.). On the other hand, discoSnp did not require any third-party tool, or any manual tuning.

Finally, this experiment showed that discoSnp scales remarkably well to large amounts of data. The KisSnp2 module required 34 h and 4.5GB of memory. The KissReads module, which assesses the average quality and coverage of the results, took 78.5 h and 5.7GB memory. In comparison, niks , KisSnp, cortex and bubbleparse exceeded the memory limit on a server with 512GB RAM.

### Evaluating discoSnp recall on a validated *Saccharomyces cerevisiae* SNP set

Using a set of biologically validated SNPs predicted from an artificial evolution study on *S. cerevisiae* (19), discoSnp recall could be evaluated on real read datasets. Among the 30 reference validated isolated SNPs, 28 were predicted by discoSnp, thus giving an estimated recall of 93.3%.

The two SNPs were correctly predicted by using the −b 2 discoSnp option that reports all bubbles including branching ones. This suggests that these SNPs may be located in complex regions of the yeast genome.

Overall, this experiment demonstrates the good performances of discoSnp at discovering SNPs from pooled samples, even those with low allelic frequencies: most of the reference SNPs were reported in the initial study with a MAF <20%. Note that no SNP with a MAF <10% was experimentally validated, so we could not assess the recall on these very low frequency SNPs.

### Use case example with experimental validation on SNPs in the *Ixodes ricinus* genome

We conducted a study on real data, including an experimental validation on SNPs selected from discoSnp predictions. This was part of a population genetic study on the tick species *I. ricinus*, the main vector species of human and animal vector-borne diseases in Europe. Given the stake of tick-borne diseases in public health (26,27), it is necessary to have an accurate description of the genetic variability within and among populations of ticks, with the aim of developing efficient control methods against this vector. To this end, highly resolutive genetic markers, like SNPs, provide particularly valuable information to estimate genetic variability and also to estimate the dispersal and genetic structure of tick populations.

No genomic resources, nor a reference genome, were available until now for this species. This study fits a case in which discoSnp is useful as (i) sequenced material exists but no reference genome is available and (ii) one is interested in detecting a small set of highly confident heterozygous SNPs. Therefore, discoSnp was applied on a 454 read set obtained from pooling and sequencing of several tick individuals isolated from natural populations (2).

discoSnp detected 321 088 SNPs of which 384 were selected, according to their minimal and maximal coverage and context sequences for experimental validation (see ‘Materials and Methods’ section). Note that as in this context there is no need to discriminate SNPs between conditions, the Phi coefficient was not used. Primers were designed for each selected SNP and 464 individuals were then genotyped for these 384 SNPs using the *Fluidigm* technology. Among them, 368 SNPs (95.8%) were retrieved with a minor allele frequency varying between 0.04 and 0.5, with a mean value of 0.23. Of the remaining 16 SNPs, 5 SNPs were not amplified and 11 presented only one of the two alleles.

## DISCUSSION

discoSnp is robust with respect to input parameters, and easy to use. The input of the software is an ordered list of any number of raw read dataset(s) (fasta or fastq, gzip compressed or not), and two parameters (*k*-mer size and minimal coverage threshold). These two parameters have limited impact on results quality (see Supplementary Additional File 1, Figures S1 and S2), as long as they are coherent with respect to the input data (read length and approximate coverage). The output is a fasta file composed of sequences containing ranked SNPs, together with their coverage and average PHRED quality.

As there exists no exhaustive and perfect list of the isolated SNPs present in a real dataset, a large part of the results discussed in this paper were obtained on simulated data. However, we paid special attention to the simulations being realistic. The use of simulated data allowed us to evaluate the correctness of our method in a controlled environment, and to analyze false positive and false negative calls. This highlighted that the main source of wrong calls is the presence of repeated sequences in the genomes.

Two distinct strategies may be adopted for distinguishing SNPs from approximate repeats. On complex genomes, which are repeat-prone, the choice of the strategy influences the results. Removing all branching bubbles (−b 0 option) is a good way to obtain high confident coverage (precision of ≈97% on human datasets) at the expense of missing some SNPs located into repeats. On the other hand, accepting simply branching bubbles, i.e. non-symmetrical (−b 1 option), enables to detect more SNPs that are located in repeated regions, but leads to an increase of the number of false positives and to the detection of non-isolated SNPs. Therefore, the choice between these two strategies is determined by the level of complexity of the genome and by the specific needs of the user.

Importantly, SNPs can be ranked according to the Phi coefficient, enabling to highlight true SNPs that discriminate conditions and to discard putative false positives due to repeats. Such feature can be precious for many biological studies, where one is often interested in finding a subset of high confidence SNPs that have opposite or only different allele frequencies between individuals or pooled samples. This was the case, for instance, when comparing two inbred mouse strains. Moreover, our simulations on a human chromosome show that, by removing low-ranked SNPs, the recall of discriminant SNPs drops only by 1.3%, while the precision increases from 92.3 to 99.7%.

A distinctive advantage of our method is its extremely low memory usage. For instance, in the mouse study cited above, nearly 3 billion reads (100 bp) were analyzed by discoSnp, using at most 5.7GB of memory. This is not at the expense of prohibitive running times, as discoSnp stays faster than all other known *de novo* SNP detection tools. discoSnp is usable on the standard desktop computer of any biological lab, enabling studies that were not possible with other available *de novo* SNP detection tools (that require at least two orders of magnitude more memory).

One limitation of discoSnp is that it cannot find the genomic locations of SNPs. However, in numerous biological applications, the localization of the polymorphisms is not required. For instance, discoSnp can be applied to identify SNPs associated to some phenotypic traits or diseases. Another limitation of discoSnp is induced by the fact that it focuses on isolated SNPs. If this kind of SNPs are well suited for designing markers, they do not represent the full SNP diversity and thus, they cannot be used directly for statistical downstream studies as phylogeny reconstruction, or estimation/comparison of the genetic diversity among or between natural populations. However, sequences obtained around those SNPs of interest can be genotyped at larger population scales with standard genotyping technologies or used for diagnostic assays. This was actually the case for the tick study, where natural populations were genotyped to characterize their reproductive mode (level of inbreeding) and to estimate the gene flow within and among populations at various spatial scales. Moreover, SNPs discovered by discoSnp and selected with respect to primer hybridization features, are currently used to build a genetic map based on the analysis of the segregation of parental alleles in the offspring of several controlled crosses.

A future development will consist in integrating *de novo* SNP, and possibly other polymorphisms such as indels and structural variants detection tools (28), with *de novo* assembly. This solution would unite the power of both approaches, facilitating the assembly by tackling the polymorphism problem and by conserving the recall and precision performances of methods such as discoSnp. This idea would lead to assembled genomes represented no more as ‘simple’ linear sequences but as graphs such as suggested by the fastg format http://fastg.sourceforge.net/, in which polymorphisms are conserved. Such a change would open the way to new possibilities of storage and use of polymorphisms.

## CONCLUSION

discoSnp is an integrated reference-free software designed to find SNPs that can be subsequently used as high-quality genetic markers. discoSnp combines several advantages: (i) robust detection and ranking of isolated SNPs, (ii) support for any number of read datasets, (iii) scaling to big data studies thanks to a highly memory efficient data structure, (iv) lower running times than other reference-free tools and (v) an easy to use software, capable of processing billions of reads from a mammalian genome with a single command.

The experiments on a dataset composed of two simulated diploids show that discoSnp provides similar results, both in terms of precision and recall, to those of other state-of-the-art reference-free SNP detection methods, while being faster and needing at least two orders of magnitude less memory. Experiments on simulated haploids show that, when analyzing together more than two individuals, discoSnp outperforms the other tools, both in terms of computational resources and results quality. Applied on real datasets, results confirm the capacity of discoSnp to scale-up to large volumes of data (less than 6GB memory on 3 billion Illumina reads), as well as its high precision, i.e. an experimental validation conducted on an arthropod species (the tick *I. ricinus*) on which *de novo* sequencing was performed, confirmed 96% of the predicted SNPs that were tested.

The discoSnp source code, available under CeCILL license, can be downloaded from http://colibread.inria.fr/discoSnp/. Moreover, this web page shows how to integrate discoSnp in any Galaxy instance using the *GenOuest Tool Shed*.

## SUPPLEMENTARY DATA

Supplementary Data are available at NAR Online.

SUPPLEMENTARY DATA
