# Supplementary material for: Reference-free detection of isolated SNPs
Source: Nucleic Acids Res. 2014 Nov 17;43(2):e11. doi: 10.1093/nar/gku1187 (PMC4333369; doi:10.1093/nar/gku1187)
Supplement: SUPPLEMENTARY DATA [file supp_43_2_e11__index.html]

Reference-free detection of isolated SNPs — Reference-free detection of isolated SNPs — SUPPLEMENTARY DATA 

# Reference-free detection of isolated SNPs

## SUPPLEMENTARY DATA

**Files in this Data Supplement:**

- SUPPLEMENTARY DATA
