# Supplementary material for: Reference-free detection of isolated SNPs
Source: Nucleic Acids Res. 2014 Nov 17;43(2):e11. doi: 10.1093/nar/gku1187 (PMC4333369; doi:10.1093/nar/gku1187)
Supplement: SUPPLEMENTARY DATA [file supp_gku1187_nar-01427-met-g-2014-File016.pdf]

Published online XX XXX 2014 *Nucleic Acids Research, 2014, Vol. XX, No. XX* **1–5**
doi:10.1093/nar/gkn000

Reference-free detection of isolated SNPs Additional File 1

Raluca Uricaru [1,2,3*], Guillaume Rizk [4], Vincent Lacroix [5,6], Elsa Quillery [7,8], Olivier Plantard [7,8], Rayan Chikhi [9], Claire Lemaitre [4*] and Pierre Peterlongo [4*]

[1]University of Bordeaux, CNRS / LaBRI, F-33405 Talence, France, [2]University of Bordeaux, CBiB, F-33000 Bordeaux, France, [3]INRA, UMR1349 IGEPP, Le Rheu, France, [4]GenScale, INRIA Rennes Bretagne-Atlantique, IRISA, Rennes, France, [5]BAMBOO, INRIA Grenoble Rhone-Alpes, Lyon, France, [6]Laboratoire de Biométrie et Biologie Évolutive, Université Lyon 1 UMR CNRS 5558, Lyon, France, [7]INRA, UMR1300 Biology, Epidemiology and Risk Analysis in Animal Health, Nantes, France, [8]LUNAM University, Oniris, Nantes Atlantic College of Veterinary Medicine and Food Sciences and Engineering, UMR BioEpAR, Nantes, France and [9]Department of Computer Science and Engineering, The Pennsylvania State University, USA

Received January 1, 2009; Revised February 1, 2009; Accepted March 1, 2009

DATA SIMULATION AND EVALUATION PROTOCOLS

Data simulation

In the main paper, we present the results for several datasets that were simulated from two distinct data sources:

- Human chomosome 1 (GRCh37/hg19 reference assembly version), ≈ 249 million base pairs. To obtain a realistic distribution of SNPs and genotypes, SNP positions are extracted from two vcf files produced by the "1000 genome project" (phase 1 release). In detail, two vcf files were retrieved from the "1000 genome project" (phase 1 release), corresponding to the human chromosome 1 of two individuals: HG00096 and HG00100. We then generated the genome sequences for the two diploids, i.e. two sequences per individual, by placing the substitutions listed in the vcf files on the human reference sequence (GRCh37/hg19 reference assembly version). In the case of a homozygous SNP, the same nucleotide was placed on the two sequences, while for a heterozygous SNP, one sequence was randomly chosen for each of the two nucleotides.
- Bacterial *Escherichia coli* K- 12, MG1655 strain, ≈ 4.6 million base pairs. From this reference sequence, we generated 30 *Escherichia coli* individuals by simulating SNPs based on a site frequency spectrum pattern, i.e. most SNPs are in one sample, half as many in two samples, a third in three samples, and so on. More precisely we introduced $X_i = \max(\frac{S}{i2^i}, 1)$ SNPs occurring in i of the 30 genomes (for i in $[1,29]$), with S being the total number of sites that were mutated, i.e. 69,600 sites in our case. SNPs were distributed uniformly along the genomes.

We then used our own read simulator, *Mutareads*, to simulate an Illumina sequencing for the two human diploids, and for the 30 bacterial individuals. The sequencing simulation was carried out by sampling equal-length reads (100 bp) from each sequence, with a uniform probability distribution, on a 40x coverage basis (2x20x for the diploid individuals). Substitution errors were uniformly distributed along each read with a fixed probability (1%).

DISCOSNP, as well as the other reference-free SNP calling tools, were run on the following datasets: one diploid individual (HG00096), the two diploid individuals (HG00096 and HG00100), two haploids (among the 30 *Escherichia coli* individuals), three haploids and so on, up to 30.

Precision and recall computation

For the tests on simulated data, both in the main paper and in this file, we provide recall and precision measures. For this purpose, we produce a *multi-fasta* file for each simulated dataset, $ref\_snps.fa$. These files are formatted as the DISCOSNP output and contain all the isolated SNPs among the complete set of generated SNPs for the given dataset. A SNP is considered as isolated if among the whole subset of considered simulated genomes, no other SNPs are simulated in the $k-1$ positions before and after the SNPs locus. They will be subsequently used as exhaustive and exact reference lists to compute precision and recall for each dataset.

More specifically, a $ref\_snps.fa$ file contains pairs of sequences, where each pair represents an isolated SNP (one sequence corresponds to one path of the SNP, and the second corresponds to the other path). Every such sequence (or path) has a $2k-1$ length, where the first $k-1$ and the last $k-1$ characters are identical between the two paths of a SNP, while the two characters placed on the kth position correspond to the mutation. Predicted SNPs are then mapped to these reference SNP sequences using GASSST (1). This enables to validate the predicted SNPs, i.e. the $2k-1$ sequences corresponding

*To whom correspondence should be adressed : ruricaru@labri.fr, claire.lemaitre@inria.fr and pierre.peterlongo@inria.fr

© 2014 The Author(s)
This is an Open Access article distributed under the terms of the Creative Commons Attribution Non-Commercial License (http://creativecommons.org/licenses/by-nc/2.0/uk/) which permits unrestricted non-commercial use, distribution, and reproduction in any medium, provided the original work is properly cited.

to the SNPs detected by DISCOSNP (or by any other tool) are mapped on the reference SNP sequences; a predicted SNP is validated if both its paths map perfectly and entirely (with 100% identity) the two paths of one of the SNPs present in this $ref\_snps.fa$ file.

Precision and recall are then computed as follows. The results of the mapping enable to compute the *true positives* (TP), i.e. predicted SNPs whose both paths are perfectly mapped on two paths of the $ref\_snps.fa$ file, and the *false positives* (FP), i.e. the other predicted SNPs. We call *false negatives* (FN) the non mapped SNPs from the reference list.

Finally, the *precision* is computed as the number of TP divided by the total number of SNPs found by DISCOSNP, while the *recall* is given as the number of TP divided by the total number of SNPs in the reference list.

In the case when more than two genomes are compared and some branchings are allowed in the bubbles, some non-isolated SNPs can be found by DISCOSNP. This happens when there exists at least two genomes (or individuals) among the compared ones with distinct genotypes for the considered SNPs but the same genotype for the other close SNPs. Such a SNP can be detected by DISCOSNP, but as we focus on isolated SNPs only, it would be considered as a false positive by our evaluation process. To compute more relevant precision values, only predictions that do not correspond to any simulated SNP (isolated or not) were considered as false positives. This was used in the main paper for the two human (chromosome 1) diploids experiment when using DISCOSNP or BUBBLEPARSE with parameters allowing for some branchings inside the bubbles (parameters -b 1 and depth=1 respectively).

HUMAN FULL RESULTS

	Tool	Parameters	Precision	Recall
★	DISCOSNP	$b=0$, $c=4$, $k=31$	96.98	71.99
★	BUBBLEPARSE	$d0$, $c=3$, $k=31$	95.78	72.71
	CORTEX	$k31$, $c=$auto	96.64	68.30
★	CORTEX	$k61$, $c=$auto	97.22	69.70
★	DISCOSNP	$b1$, $c=4$, $k=31$	92.3*(88.10)	79.22
★	BUBBLEPARSE	$d1$, $c=3$, $k=31$	91.66*(87.47)	76.60
	BUBBLEPARSE	$d2$, $c=3$, $k=31$	86.20* (81.88)	79.80
	BUBBLEPARSE	$d3$, $c=3$, $k=31$	78.70* (74.51)	82.20
	hybrid strategy	unfiltered	95.50	72.91
★	*hybrid* strategy	filtered	96.18	72.86

Table 1. Results obtained by various tools with various parameters on human chromosome 1 dataset composed of two individuals (described in Section "*Data simulation*"). Lines starting with ★ were already indicated in the manuscript. * denotes that precision was computed by considering as false positives only bubbles that do not correspond to any simulated SNP (isolated or not). Note that $c=3$ for BUBBLEPARSE and $c=4$ for DISCOSNP are equivalent as they filter out data seen three times or less.

In the paper only best results for each tested tool are presented. The Table 1 presents the full obtained results. The *hybrid* strategy (SOAP+bowtie2+GATK) provides SNPs with low coverage, which mainly correspond to false positives. As DISCOSNP and other tested tools filter out low covered k-mers (seen less than 4 times in each dataset), we decided to apply the same filtering for the *hybrid* strategy, i.e. filtering out SNPs covered less than four times. The command lines that were used for the different tools are indicated in Table 2.

DETAILS ABOUT THE SACCHAROMYCES CEREVISIAE EXPERIMENT

Preparing the data

The 24 read sets were downloaded from the NCBI Sequence Read Archive (with the accession number SRA054922). Read pairs were separated using the *fastq-dump* command from *sra-toolkit*, with the *–split-3* option. The SRA read file names are detailed in Table 3. Read sets were prepared applying the protocol from the Kvitek study (2). The obtained coverages range from 348x to 1536x (see Table 4) depending on the experiment.

Table 3. SRA read file names associated to their respective experiment in (2) study. Each value corresponds to the SRR file name. For instance, 515969 corresponds to SRR515969.sra file.

	Generation							
	007	070	133	196	266	322	385	448
E1	515969	519088	519089	519090	519091	519092	519093	519094
E2	515482	515483	515484	515966	515485	515486	515967	515968
E3	515487	515488	519064	519081	519084	519085	519086	519087

We extracted the set of isolated SNPs among the validated ones in (2). We generated the set of associated bubbles in fasta format using the S288c reference sequences from the Saccharomyces Genome Database (`http://www.yeastgenome.org/`). These bubbles were used as a reference for estimating DISCOSNP recall.

Table 4. Coverage per generation and per population (average per value on both files of the pair). RC stands for "Read Coverage", and it is computed as $\sum \text{read sizes} \times \frac{1}{\text{genome size}}$. KC stands for "$k$-mer coverage", and it is computed as $\frac{\sum \text{read sizes} - \text{number of reads} \times (k-1)}{\text{genome size}}$.

	Generation							
	007	070	133	196	266	322	385	448
E1 RC	797	1061	1142	1138	1209	1062	1478	1536
E1 KC	423	609	649	646	715	621	945	981
E2 RC	348	407	369	396	479	505	458	619
E2 KC	184	217	194	208	260	278	258	359
E3 RC	763	704	636	756	829	736	636	868
E3 KC	409	391	349	423	455	419	341	500

Running DISCOSNP

DISCOSNP was run independently on the three populations E1, E2 and E3, with defaults parameters, except for the c value that was fixed to 11. The $c=11$ value was chosen with respect to minimal k-mer coverage observed in the data (see Table 4). A first experiment was performed with -b 1 option and another experiment with -b 2. For each experiment, the 16 (8x2) read sets were used collectively. For instance the E1 experiment was performed using the following command line:

```
./run_discoSnp.sh -b 1 -c 11 -r
"E1_gen007_forward.fq.gz E1_gen007_reverse.fq.gz
E1_gen070_forward.fq.gz E1_gen070_reverse.fq.gz
E1_gen133_forward.fq.gz E1_gen133_reverse.fq.gz
E1_gen196_forward.fq.gz E1_gen196_reverse.fq.gz
E1_gen266_forward.fq.gz E1_gen266_reverse.fq.gz
E1_gen322_forward.fq.gz E1_gen322_reverse.fq.gz
E1_gen385_forward.fq.gz E1_gen385_reverse.fq.gz
E1_gen448_forward.fq.gz E1_gen448_reverse.fq.gz"
```

ANALYSIS OF THE ROBUSTNESS OF DISCOSNP RESULTS

Simulation protocol

This section proposes additional results on simulated datasets, when varying the sequencing simulation process (read

DISCOSNP

```
./run_discoSnp.sh -r "individualHG00096_reads.fasta.gz individualHG00100_reads.fasta.gz" -k 31 -c 4 -p res_human
```

BUBBLEPARSE

```
/bin/cortex_bub_31 -k 31 -n 26 -b 70 -c 100 -s 3 -i individualHG00096_reads.fasta -t fasta -o human1.ctx
/bin/cortex_bub_31 -k 31 -n 26 -b 70 -c 100 -s 3 -i individualHG00100_reads.fasta -t fasta -o human2.ctx
echo "human1.ctx 0"  > ctxfiles.txt
echo "human2.ctx 1"  >> ctxfiles.txt
/bin/cortex_bub_31 -k 31 -n 26 -b 70 -w $depth,41 -c 100 -s 3 -i ctxfiles.txt -t binary -f snpout
echo "EXPECTEDCOVERAGE \"0,10,100,0\"" > bpoptions.txt
echo "EXPECTEDCOVERAGE \"1,10,100,0\"" >> bpoptions.txt
echo "MINIMUMCONTIGSIZE \"100\"" >> bpoptions.txt
./bin/bubbleparse_31 -f snpout -t table.txt -c table.csv -r table.fa -k 31 -o bpoptions.txt -x -d log.txt
```

CORTEX

```
perl ../../CORTEX_release_v1.0.5.21/scripts/calling/run_calls.pl --first_kmer 31 --last_kmer 61 --kmer_step 30 \
 --fastaq_index INDEX --auto_cleaning yes --bc yes --pd no --outdir "cortex_2humans_no_ref" \
 --outvcf "cortex_2humans_no_ref" --ploidy 2 --genome_size 249250621  \
 --stampy_bin /mnt/cbib/read2snps/stampy-1.0.23/stampy.py --mem_height 25 --mem_width 100  \
 --vcftools_dir /mnt/cbib/read2snps/vcftools_0.1.9/ --do_union yes --ref Absent \
 --workflow joint --logfile log.txt,f
```

Hybrid approach

```
#Soap and filtering
SOAPdenovo-63mer pregraph -s soap.config  -o soapNARHUM -K 31 -d 5
SOAPdenovo-63mer contig -g soap NARHUM
python filter_fasta_by_length.py soapNARHUM.contig NARHUM_ref.contigs.fa 100
#bowtie
bowtie2-build -f NARHUM_ref.contigs.fa NARHUM_ref.contigs.fa_index  ;
bowtie2 -f --non-deterministic --threads 8  --rg-id "readsnp" --rg "SM:readsnp" --rg "PL:Illumina" \
 --rg "LB:simumima" -x NARHUM_ref.contigs.fa_index  -U individualHG00096_reads.fasta.gz \
 |  samtools view -bS - >  NARHUM_bw2.bam;
bowtie2 -f --non-deterministic --threads 8  --rg-id "readref" --rg "SM:readref" --rg "PL:Illumina" \
 --rg "LB:simumima" -x NARHUM_ref.contigs.fa_index  -U individualHG00100_reads.fasta.gz \
 |  samtools view -bS - >   NARHUM_bw2r.bam;
#GATK analysis
java -Xmx4g -jar ./CreateSequenceDictionary.jar  R=NARHUM_ref.contigs.fa  O=NARHUM_ref.contigs.dict
java -Xmx8g -jar GenomeAnalysisTK.jar \
 -R NARHUM_ref.contigs.fa \
 -T UnifiedGenotyper \
 -glm SNP \
 -I NARHUM_bw2.bam \
 -I NARHUM_bw2r.bam \
 -o NARHUM_snp.vcf
```

Table 2. Command lines for DISCOSNP, BUBBLEPARSE, CORTEX and for the *hybrid* approach, which were used on the human dataset described in Section “*Data simulation*”. However, some of BUBBLEPARSE scripts needed further manual tuning in order to be run.

simulator and sequencing coverage), when varying the density and the repartition of the SNPs, and when varying the DISCOSNP main parameters (k and c).

The first dataset contains two *E. coli* individuals simulated as explained in Section “*Data simulation and evaluation protocols*” (100bp long Illumina reads with uniformly distributed errors, for a 40x coverage).

The second dataset was simulated from the human chromosome 1 (hg19 assembly), by generating a mutated sequence in which only uniformly distributed homozygous SNPs were simulated at 0.1% SNP rate. Sequencing of both the reference and the mutated sequence was then simulated for a 50x coverage. Note that the simulations made on the human sequence are distinct from those presented in the paper, thus explaining why results are slightly different from those presented in the paper with same parameters.

Results when varying the simulation parameters

Influence of the read simulation method

Knowing that SNP detection methods can be misled by sequencing errors, as they potentially generate false positives, we checked the robustness of DISCOSNP with respect to the read simulator. Besides *Mutareads*, five simulation tools were tested: *Art* (4), *GemSim* (5), *Metasim* (6), *SimSeq* (7), and *WGSim* from the *Samtools* package (8), each of them implementing a specific error profile. The six simulators were applied on the two *E. coli* individuals, as described in Section “Simulation protocol”. Results are summarized in Table 5. These results show that, except for *Art*, DISCOSNP produces similar results regardless the simulation tool. Poor results in *Art* case are most certainly due to its high error rate, i.e. it simulates more than six errors per read on average, which is not consistent with what is happening in real data. Moreover, *GemSim* was, in average, 100 times slower than the other read simulators, thus making it unusable on large datasets such as human data.

For the human experiment, due the previously exposed reasons, we excluded *Art* and *GemSim* simulators. Once again,

Table 5. DISCOSNP results (with $c=4$ and $k=31$), on data simulated from the *E. coli* dataset with several read simulators.

Read simulator	Recall (%)	Precision (%)
Art	0	33.3
Gemsim	98.6	98.7
Metasim	97.6	98.1
Mutareads	98.6	97.7
SimSeq	98.8	98.8
WGSim	98.6	98.7

results in Table 6 show that DISCOSNP produces similar results regardless the simulation method.

Table 6. DISCOSNP results (with $c=5$ and $k=31$), on data simulated from human chromosome 1 with several read simulators.

Read simulator	Recall (%)	Precision (%)
Metasim	87.1	90.3
Mutareads	86.6	90.5
SimSeq	87	90.6
WGSim	87	90.5

As we showed that DISCOSNP results are independent of the read simulator that is used, we chose *Mutareads* to perform the simulation experiments presented in the paper and in this file, as it is the fastest among the tested simulators.

Influence of the sequencing depth

In order to estimate the effect of the read coverage on DISCOSNP results, we performed simulations with *Mutareads* on the human chromosome 1 sequence by using a range of coverage values. The results presented in Table 7 show that the read coverage influences the result quality. As expected, the higher the read coverage, the better the precision/recall results. However, these results suggest that even for coverage values as low as 20x, DISCOSNP calls 79.5% of SNPs, while maintaining a good precision (89.9%).

Table 7. DISCOSNP precision and recall results on simulated datasets generated from human chromosome 1, with increasing sequencing depths (using $k=31$).

Coverage	min coverage value (c)	Recall %	Precision %
10x	1	65.9	73.7
20x	2	79.5	89.9
30x	3	84.5	90.3
40x	4	86.0	90.4
50x	5	86.6	90.5

Influence of the SNP density

In order to estimate the influence of the SNP density on DISCOSNP results, we generated datasets with densities varying from 0.06% (based on (9)), to 0.1%, and to to an over-estimation of 1% of SNPs.

In the results presented in Table 8, precision varies from 85.8% to 98.3%. The higher the SNP density, the higher the precision. This reveals that the number of false positives is stable regardless the SNP simulation method (19088±2%), while the number of true positives grows linearly with the number of isolated SNPs.

SNP density	SNP number		DISCOSNP	
	all	isolated	Precision	Recall
1%	2,253,177	1,248,158	98.3%	84.8%
0.1%	224,876	211,492	90.5%	86.6%
0.06%	138,383	133,621	85.8%	86.7%

Table 8. DISCOSNP results on uniformly simulated SNP sets, with different SNP densities, from the human chromosome 1

Results when varying DISCOSNP parameters

Influence of the k value

Any algorithmic method based on *de Bruijn graphs* is highly dependent on the k-mer size. In order to analyze the influence of the k-value on DISCOSNP results, we performed an experiment on human chromosome 1. The results that are shown in Figure 1 were produced with DISCOSNP for k-values going from 15 to 45.

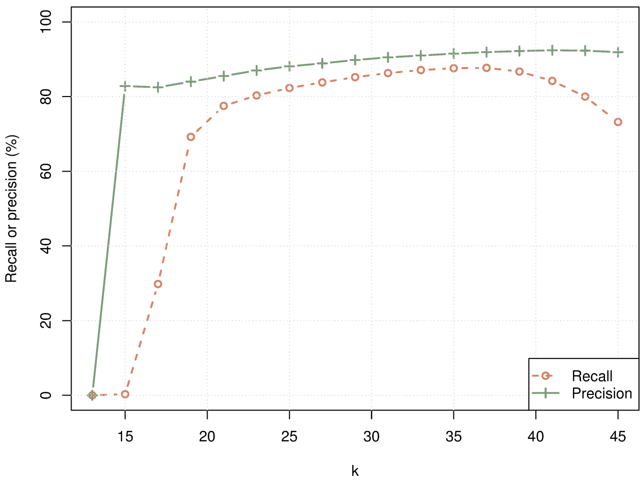

Figure 1. DISCOSNP recall and precision results on the human chromosome 1 dataset, for increasing k values and with $c=5$.

These results confirm that the k-value influences the quality of the results. For small k-values, due to the increasing number of branching k-mers, a larger number of branching bubbles are discarded, which leads to a low recall. Precision is also affected as there is an increasing number of bubbles that are generated by inexact repeats. Indeed, an inexact repeat of length $2k-1$ generates a bubble, and the frequency of such repeats in the genomes, increases when k decreases. On the other hand, the recall decreases for large k values ($\gtrsim 37$), as there are fewer reads that overlap on at least k characters.

With current NGS reads, a good trade-off value for k is ≈ 31. As presented in Figure 1, the best precision/recall is reached with $k=37$. However, values that are larger or equal to 32 are usable at the cost of either larger RAM consumption or longer data structure creation times. This study also shows that, even if the k value influences DISCOSNP results, any choice of k between 25 and 39 provides high quality results (with precision varying from 88.1% to 92.2% and recall varying from 82.3% to 86.7%).

Influence of the minimal coverage value

DISCOSNP offers the possibility to filter out k-mers whose number of occurrences in all read set is below a user-defined threshold, thus enabling to discard k-mers that are most probably due to sequencing errors. The results that are presented in Figure 2 are obtained with DISCOSNP ($k=31$) on the 50x dataset simulated from human chromosome 1.

These results show that for large minimal coverage values (≥ 6), recall decreases due to low covered SNPs, while precision slightly decreases as the proportion of SNPs due to inexact repeats increases. With no filter ($c=1$), sequencing errors are not filtered out, and a high ratio of the reported SNPs are due to sequencing errors. Moreover, recall is slightly poorer as the graph is more complex, and more bubbles are discarded by DISCOSNP (as they are branching).

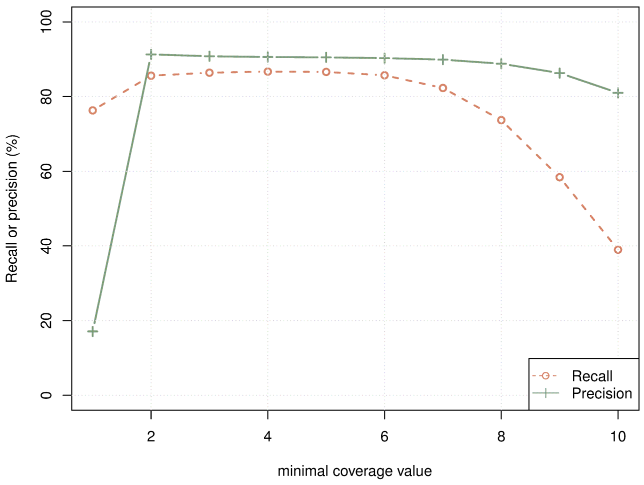

Figure 2. DISCOSNP recall and precision results on the human chromosome 1 dataset, with respect to the minimal coverage value.

RUNNING TIMES FOR THE MULTIPLE INDIVIDUALS STUDY

Here, we present the comparative running times of DISCOSNP, CORTEX and the hybrid approach when analyzing an increasing number of read sets. The results, presented in Figure 3, were obtained in the study concerning the 30 haploid datasets simulated from E. coli. They show that running times grow linearly with respect to the number of individuals regardless the method, and that DISCOSNP runs faster than the two other methods.

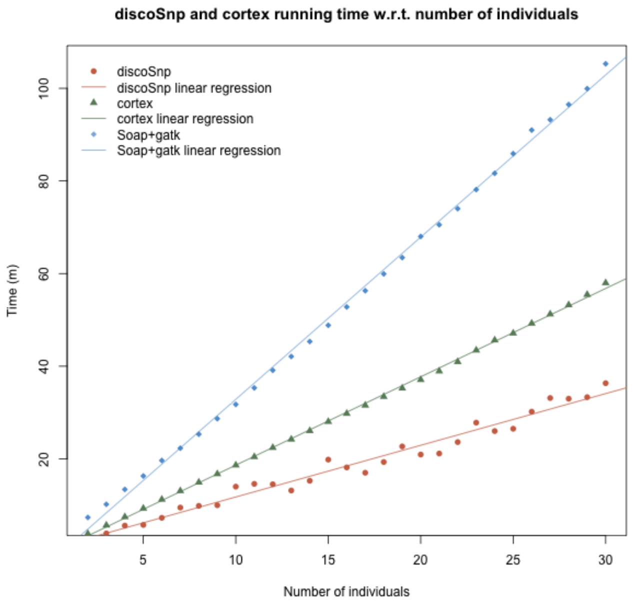

Figure 3. Running times of the hybrid Soap+gatk method, of CORTEX and of DISCOSNP, when analyzing an increasing number of read sets from 2 to 30.

REFERENCES

1. Rizk, G. and Lavenier, D. August 2010 *Bioinformatics* **26(20)**, 2534–2540.
2. Kvitek, D. J. and Sherlock, G. November 2013 *PLoS genetics* **9(11)**, e1003972.
3. Martin, M. (2011) *EMBnet.journal* **17**, 10.
4. Huang, W., Li, L., Myers, J. R., and Marth, G. T. February 2012 *Bioinformatics (Oxford, England)* **28(4)**, 593–4.
5. McElroy, K. E., Luciani, F., and Thomas, T. January 2012 *BMC genomics* **13(1)**, 74.
6. Richter, D. C., Ott, F., Auch, A. F., Schmid, R., and Huson, D. H. January 2008 *PloS one* **3(10)**, e3373.
7. Earl, D., Bradnam, K., St John, J., Darling, A., Lin, D., Fass, J., Yu, H. O. K., Buffalo, V., Zerbino, D. R., Diekhans, M., Nguyen, N., Ariyaratne, P. N., Sung, W.-K., Ning, Z., Haimel, M., Simpson, J. T., Fonseca, N. a., Birol, I., Docking, T. R., Ho, I. Y., Rokhsar, D. S., Chikhi, R., Lavenier, D., Chapuis, G., Naquin, D., Maillet, N., Schatz, M. C., Kelley, D. R., Phillippy, A. M., Koren, S., Yang, S.-P., Wu, W., Chou, W.-C., Srivastava, A., Shaw, T. I., Ruby, J. G., Skewes-Cox, P., Betegon, M., Dimon, M. T., Solovyev, V., Seledtsov, I., Kosarev, P., Vorobyev, D., Ramirez-Gonzalez, R., Leggett, R., MacLean, D., Xia, F., Luo, R., Li, Z., Xie, Y., Liu, B., Gnerre, S., MacCallum, I., Przybylski, D., Ribeiro, F. J., Yin, S., Sharpe, T., Hall, G., Kersey, P. J., Durbin, R., Jackman, S. D., Chapman, J. a., Huang, X., DeRisi, J. L., Caccamo, M., Li, Y., Jaffe, D. B., Green, R. E., Haussler, D., Korf, I., and Paten, B. December 2011 *Genome research* **21(12)**, 2224–41.
8. Li, H., Handsaker, B., Wysoker, A., Fennell, T., Ruan, J., Homer, N., Marth, G., Abecasis, G., and Durbin, R. August 2009 *Bioinformatics (Oxford, England)* **25(16)**, 2078–9.
9. Sachidanandam, R., Weissman, D., Schmidt, S. C., Kakol, J. M., Stein, L. D., Marth, G., Sherry, S., Mullikin, J. C., Mortimore, B. J., Willey, D. L., Hunt, S. E., Cole, C. G., Coggill, P. C., Rice, C. M., Ning, Z., Rogers, J., Bentley, D. R., Kwok, P. Y., Mardis, E. R., Yeh, R. T., Schultz, B., Cook, L., Davenport, R., Dante, M., Fulton, L., Hillier, L., Waterston, R. H., McPherson, J. D., Gilman, B., Schaffner, S., Van Etten, W. J., Reich, D., Higgins, J., Daly, M. J., Blumenstiel, B., Baldwin, J., Stange-Thomann, N., Zody, M. C., Linton, L., Lander, E. S., and Altshuler, D. February 2001 *Nature* **409(6822)**, 928–33.
